# Probabilities

**Published:** 1877-07

**Authors:** B. Browne


### PROBABILITIES.

B. BROWNE.

Taking up the morning paper, one of the first articles of interest to which every one is accustomed to turn, is the report of our govenment prognosticator of the weather. He settles and unsettles the proposed plans of the day in a surprising manner. We have learned to place our dependence upon him—he is a "good thing" and we all know it.

His process of reasoning is very simple but comprehensive, for he gathers up the reports sent to him from the four quarters of these United States, notes the progressive changes of wind and temperature from one quarter as opposed to counter-currents of the others. These he balances with a skillful knowledge, and proclaims to us through the morning "Daily."

As we said, he is a good thing. He tells us to look out for storms, and though the sun shines brightly we take our umbrella from the rack as we go over to town to business, believing we shall need its protection on our return.

This great progress in science and the utilizing of the laws of probabilities in the atmospheric changes each day suggests a new field of research in "probabilities." Having succeeded so well in so trivial a matter as telling us "it's going to rain," why ought any surprise to be excited if we inquire, shall we not have a "domestic weather probabilities?" Some one to tell us to prepare our umbrellas which shall save us from the discomfort of a domestic shower? How nice to have the state of the weather in Bridget's domain, reported to some secluded apartment, that it may be compared with "how she blows" from Biddy, the cook! How nice to know whether it is hotter in the nursery than in the kitchen—whether all was serene when John said good-night to her across the garden gate, or whether the clouds began to gather then, to burst upon the household at seven o'clock in the morning, and no fire in the range—whether the muttering storm will pass off with heat lightning, or will a dreary storm shut down upon us, as exhilarating as an old fashioned New England nor' easter.

Then, too, 'twould be nice to have those "areas of rain" defined. Do the little puffs of wind which our better-half breathes forth, indicate a gathering breeze which may bring us from the sea of difficulties, a storm as fearful as the one the good Elijah saw gathering from a cloud no bigger than a man's hand. When a woman has a hand in that cloud, must we not beware and learn in good time what is coming? How pleasant it would be to know that these matrimonial breezes will go down as the hour passes on and sunshine dry the sprinkle-drops when first they fall—to have it put to us in plain print so that we can go to the club on an evening and not "catch it" on returning home!

Are these vain delusive longings? Cannot these pressures in the air of the household be reported in good time, so that a man may shape his plans to weather it through without danger of spoiling his good clothes in an unexpected storm? It must be some time, we are confident. Shall our generation see it? Who can say? I hope the suggestion may find favor at the Department Office and prove that there is progress in domestic science. Are we to live along as ignorant as our great grandparents were? Can't a man know "what is up" at home without putting his head into a storm-cloud? Shall Bridget rain (reign) and no one know it? Shall a freezing coldness come upon the temper of madam and we all unwittingly meet her with no comforting length of ulster to protect us?

These are only hints and suggestions thrown out in the hope that some knowing one will devote time, money and patience beyond what we possess, in a scientific investigation of this subject. There is a call for just this range of talent. Some one should be at it already.

